# Editorial: Designing and evaluating digital health interventions

**DOI:** 10.3389/fdgth.2025.1612380

**Published:** 2025-05-08

**Authors:** Kim Bul, Nikki Holliday, Edith Talina Luhanga

**Affiliations:** ^1^ Centre for Intelligent Healthcare, Coventry Universitry, Coventry, United Kingdom; ^2^ Centre for Healthcare and Communities, Coventry University, Coventry, United Kingdom; ^3^ Carnegie Mellon University Africa, Kigali, Rwanda

**Keywords:** digital, health, development, evaluation, interventions, technologies

**Editorial on the Research Topic**
Designing and evaluating digital health interventions

## Background

The World Health Organization (1) defines digital health as “*the systematic application of information and communications technologies, computer science, and data to support informed decision-making by individuals, the health workforce, and health systems, to strengthen resilience to disease and improve health and wellness*”. Although the COVID-19 pandemic accelerated development of DHIs internationally, there remains a divide between high-income countries showing a wealth of personalized and immersive health platforms while initiatives across low-and-middle income countries (LMICs) are still limited given their priority on mobile technologies and wireless connectivity due to limited infrastructure, resources and focus on basic healthcare needs (2). Hence, the academic literature is often split between examining effectiveness of these interventions in high-income countries vs. LMICs. Additionally, there is consensus that there is disparity in DHI access across groups such as women, migrants and older people (3). Despite a greater focus on women's health (e.g., perinatal mental health) and gender-specific DHIs, their healthcare is hindered due to unequal access and usage (4, 5). The Medical Research Council Framework for Complex Interventions stresses that well-designed interventions should consider theoretical frameworks of behaviour change, human-centred design through patient/public involvement and engagement (PPIE) as well as co-design, usability, feasibility and robust evaluation through trials to prove effectiveness in improving people's health (6). In response to these global disparities and methodological challenges, the current Research Topic aims to advance the field by showcasing rigorous and inclusive approaches to the design and evaluation of DHIs.

**Figure 1:**
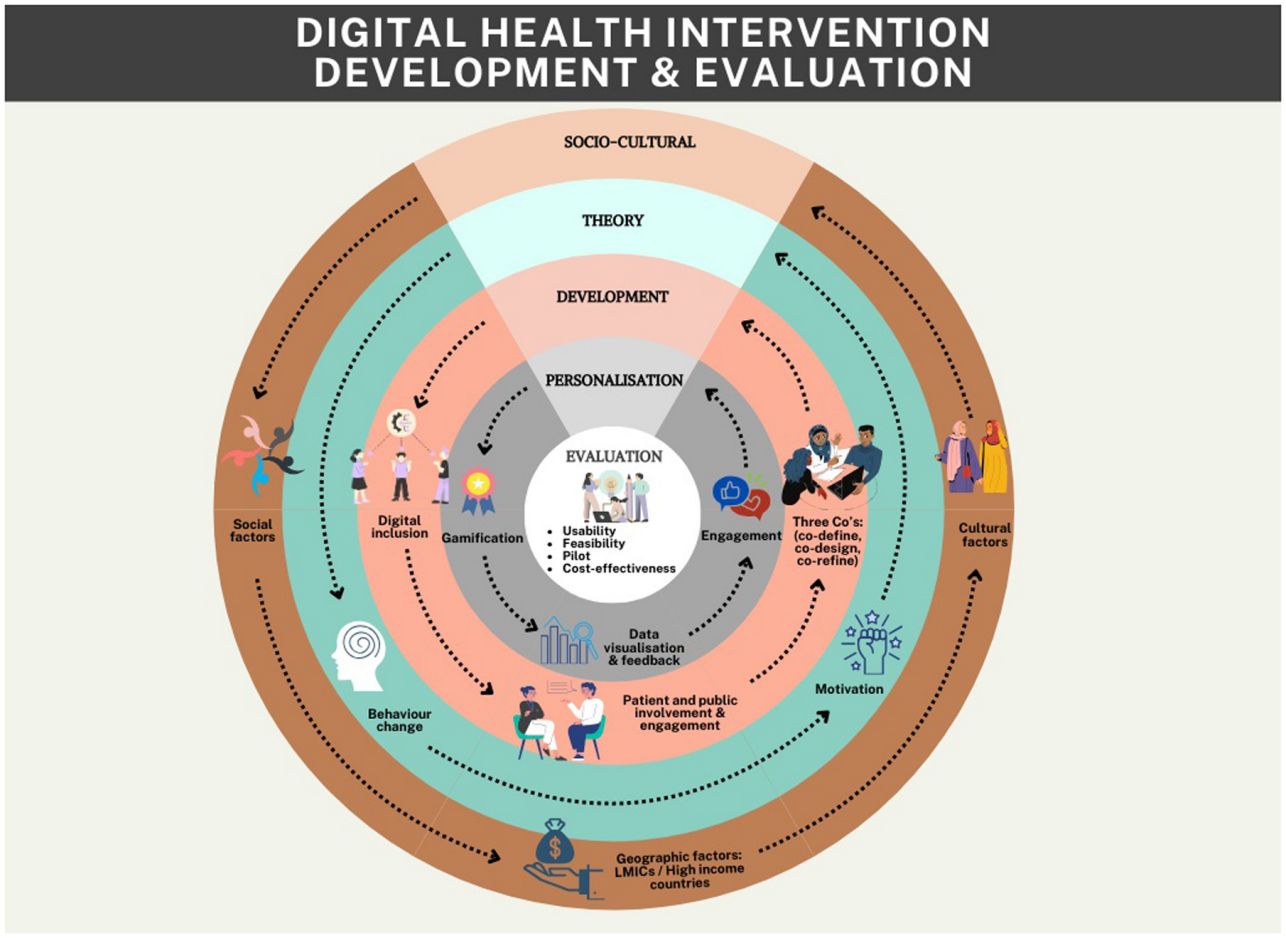
Relevant research domains in digital health intervention development and evaluation.

### Aims and objectives

The aim of the current Research Topic was to establish a collection of high-quality diverse manuscripts representing the dynamic academic field of DHIs. Scientific evidence on DHIs is continuously evolving due to its importance and relevance while there often is a lack of funding, methodological guidance and inclusivity of the digital healthcare landscape. With two systematic reviews, eight original articles, two brief research reports, one study protocol, and one curriculum, instruction and pedagogy article the current collection gives an up-to-date, multidisciplinary and widespread contribution to the field of DHIs, specifically in the area of intervention design and evaluation.

### The current research topic

Synthesizing the results of 30 RCTs, small to moderate effects of DHIs on mental health were demonstrated by Morello et al. However, due to the complexity of interventions it was not clear what the effective ingredients (e.g., enhancing cognitive reappraisal) of the interventions were. To promote better reporting of complex interventions and to support identification of working mechanisms it is recommended that interventions are described using the TIDieR checklist (7). Furthermore, to design a theoretically sound and effective DHI it is important to incorporate behaviour change strategies (e.g., self-monitoring). The brief research report from Ghantasala et al. explores tailored motivation messages as a strategy to increase engagement and physical activity. The report indicates that personalized messages adjusted to mood, self-efficacy and progress are perceived as more motivating compared to generic messages. Lakha et al. used a case study to demonstrate how a digital scrapbook format can be a feasible and accessible format for patients with chronic pain and illness. Increasing engagement across DHIs is important, as dropouts can be high [up to 82% (8)]. Gamified approaches are used as an engaging way to increase knowledge and health outcomes. In the case of the pilot study reported by Seaver et al. a game-based learning approach was used to educate people about sleep hygiene and improve outcomes such as sleep quality and anxiety. The digital therapeutics mobile app from Jeong et al. demonstrated a gamified approach to facilitate breath control. This included machine learning and biofeedback visualization to improve engagement.

Given research funding is often limited it is important to examine cost-effective ways to design and evaluate DHIs in a research context and potentially collaborate with industry initiatives. Instead of developing DHIs from scratch there are opportunities to repurpose existing interventions to improve health outcomes (9). The study from Chen et al. examined a mobile health intervention called MyTrack + - paired with existing commercials apps and weight scales—to support long-term weight loss maintenance. Small usability and pilot studies were performed to iteratively incorporate end-user feedback while keeping R&D processes efficient. For DHI design, it is important to follow established frameworks like MRC or co-design frameworks such as the three Co's framework (10, 11) to ensure the intervention fits with end-user needs. The study of Hamaguchi et al. focuses on a novel smartphone app to support patients with mild cognitive impairment and dementia. Although initial positive results were found in terms of engagement, further multi-centre randomized controlled trials are recommended to evidence improvement of other health outcomes (e.g., cognition). The study protocol developed by Castelnuovo et al. describes a clinical RCT to demonstrate efficacy of an innovative digital therapy to promote weight loss in patients with obesity by increasing their treatment adherence. Complementing trial data with process evaluation, as done by Ali et al., is crucial to gain a deeper understanding of participant experiences on how, why and when people are engaging with these tools. However, it must be noted that the field of DHIs lacks methodological guidance on evaluating its effectiveness beyond RCTs (12) and is quite segmented due to its inter-disciplinary nature and approach. Implementation science is an important aspect of DHIs and goes beyond the development of guidelines on how to implement interventions in clinical practice, for example through educating future healthcare professionals on this. The curriculum, instruction and pedagogy article from Loizou et al. uses Virtual Reality simulations for healthcare professionals to practice carer and patient interactions based on affective intelligent agents incorporated into the learning scenarios.

Healthcare access remains a global challenge and DHIs, such as telehealth, can support in streamlining provision and reducing waiting times (13). The study of Tennankore et al. describes a pilot study examining the potential of the Virtual Hallway platform in facilitating patient-focused specialist care through synchronous phone conversations. This demonstrated high acceptability and potential to reduce waitlists and unnecessary referrals. The qualitative case study approach from Sowon et al. refers to a “community of purpose” and how they affect the usage of mobile health interventions in the context of maternal care in Sub-Saharan Africa context. Although results may not be generalisable to high-income countries, it provides insights into the complexities of promoting use and adoption of DHIs across similar economic and cultural contexts. Another important topic regarding healthcare access in the field of DHIs is the topic of digital exclusion. The systematic review of Udenigwe et al. focussed on gender transformative approaches in mobile health interventions for maternal health in sub-Saharan Africa, primarily consisting of text-based messaging. It stresses the limited extent of such approaches and highlights the need for inclusivity in the digital landscape in maternal health across LMICs. Inclusivity should be considered for any DHIs across any context and research stage, including high-income countries (14). The brief research report article from Collombon et al. focuses on recruiting adults aged 50 years and older with low socioeconomic status for participation in online physical activity interventions. As stressed by the report, personal paper-based invitation letters worked best compared to social media and advertisements through a gym. It is important to use inclusive recruitment strategies to maximize diversity and ensure equal access to the research study, specifically when the end-users are hard to reach.

### Future research recommendations

The field of DHIs is developing rapidly with known potential to tackle healthcare challenges. However, challenges remain in terms of accessibility, engagement and evaluation methodologies across different patient populations and economic settings. The contributions in this collection demonstrate the importance of incorporating behavioural science, human-centered design, and rigorous evaluation frameworks to enhance effectiveness. Inclusivity must be prioritized from intervention design to recruitment strategies, to ensure equitable access for underserved groups. Interdisciplinary collaborations between academia, industry and healthcare providers are crucial and future research should explore cost-effective and scalable solutions, leveraging AI, machine learning, and gamification while maintaining a focus on ethical considerations and end-user needs. The current research topic presents a global and interdisciplinary perspective, furthering our understanding of the field and shaping future research directions and recommendations (Figure 1).

Dr. Kim Bul

Assistant Professor, Research Centre for Intelligent Healthcare, Coventry University

Nikki Holliday

Design Manager, Research Centre for Healthcare and Communities, Coventry University

Dr. Edith Talina Luhanga, Carnegie Mellon University Africa
